# Prevalence of burnout among GPs: a systematic review and meta-analysis

**DOI:** 10.3399/BJGP.2021.0441

**Published:** 2022-02-22

**Authors:** Christo Karuna, Victoria Palmer, Anthony Scott, Jane Gunn

**Affiliations:** Monash Business School, Monash University, Melbourne.; Department of General Practice, Melbourne Medical School, University of Melbourne, Melbourne.; Applied Economic and Social Research, Faculty of Business and Economics, Melbourne Institute, University of Melbourne, Melbourne.; Faculty of Medicine, Dentistry and Health Sciences; chair of primary care research, Department of General Practice, University of Melbourne, Melbourne.

**Keywords:** burnout, professional, family medicine, family physicians, family practice, general practice, general practitioners

## Abstract

**Background:**

Burnout is a work-related syndrome documented to have negative consequences for GPs and their patients.

**Aim:**

To review the existing literature concerning studies published up to December 2020 on the prevalence of burnout among GPs in general practice, and to determine GP burnout estimates worldwide.

**Design and setting:**

Systematic literature search and meta-analysis.

**Method:**

Searches of CINAHL Plus, Embase, MEDLINE, PsycINFO, and Scopus were conducted to identify published peer-reviewed quantitative empirical studies in English up to December 2020 that have used the Maslach Burnout Inventory — Human Services Survey to establish the prevalence of burnout in practising GPs (that is, excluding GPs in training). A random-effects model was employed.

**Results:**

Wide-ranging prevalence estimates (6% to 33%) across different dimensions of burnout were reported for 22 177 GPs across 29 countries were reported for 60 studies included in this review. Mean burnout estimates were: 16.43 for emotional exhaustion; 6.74 for depersonalisation; and 29.28 for personal accomplishment. Subgroup and meta-analyses documented that country-specific factors may be important determinants of the variation in GP burnout estimates. Moderate overall burnout cut-offs were found to be determinants of the variation in moderate overall burnout estimates.

**Conclusion:**

Moderate to high GP burnout exists worldwide. However, substantial variations in how burnout is characterised and operationalised has resulted in considerable heterogeneity in GP burnout prevalence estimates. This highlights the challenge of developing a uniform approach, and the importance of considering GPs' work context to better characterise burnout.

## INTRODUCTION

GP burnout (including physicians and other medical specialties) is a recognised healthcare problem that has become widespread over time and for which the adverse effects on clinicians^1^^–^^13^ and patients^2^^,^^14^ have been documented. Given these deleterious effects, estimating the prevalence of GPs’ burnout is important. Burnout is generally referred to as an inability to cope with chronic psychological stress at work because of insufficient resources to cope with job demands.^15^^,^^16^ Researchers have denoted that burnout captures three dimensions/subscales: emotional exhaustion, cynicism/depersonalisation, and personal accomplishment.^17^^–^^19^

This characterisation of burnout is also used in health care, as is aptly captured in the World Health Organization’s 11th revision of the International Classification of Diseases (https://icd.who.int). GP tasks are related to treating illness in the context of the patient’s life, belief systems, and community (thus it is person focused rather than disease focused),^20^^,^^21^ and working with other healthcare professionals to coordinate care and make efficient use of health resources.^22^^,^^23^ Although surveys on physician burnout in the US conducted by other researchers have reported that physician specialties that frequently deal with patients and their families, such as GPs, experienced considerably higher burnout rates than other specialties, it is unclear how prevalent GP burnout is.^12^^,^^24^

This systematic review aimed to conduct a synthesis of the evidence on the prevalence of GP burnout documented in the literature. In doing so it aimed to deliver a baseline picture of burnout in the GP context to establish the burden GP burnout imposes on the healthcare system. This, in turn, may benefit policymakers, healthcare institutions, clinicians, researchers, and the public to develop interventions to address the syndrome. This is especially important in the post-COVID-19 environment, which has witnessed considerably greater burden placed on GPs via more frequent patient visits and other requirements.

**Table 1:** How this fits in

GP burnout is widely recognised as a problem in health care. However, to the authors’ knowledge, no study has been conducted on the global burden of this condition. The systematic review and meta-analysis conducted show that moderate to high levels of burnout exist worldwide. However, a challenge to policymakers is the wide variation in burnout estimates across studies and countries documented in this review. The findings from this review highlight that the context within which GPs work should be considered in better understanding GP burnout.

## METHOD

### Data sources and searches

The search strategy for this systematic review was conducted using a combination of keywords and subject headings to include two concepts: ‘general practice *or* GP’ and ‘burnout’. Primary care physicians typically include GPs as well as other physicians such as paediatricians, emergency physicians, and internal medicine specialists. However, this study focuses specifically on physicians who typically undertake generalist patient care such as GPs, and excludes the other subspecialties of primary care.

Only studies that reported prevalence estimates on GP burnout in general practice using the Maslach Burnout Inventory — Human Services Survey (MBI-HSS) were included in this review. Although different burnout scales have been used in prior research, the MBI-HSS was used in this review to allow comparisons in burnout prevalence estimates across studies. Moreover, the MBI-HSS is the most widely used burnout instrument in the literature that measures burnout by capturing the different dimensions of burnout that have been identified in the literature, namely, emotional exhaustion, depersonalisation, and personal accomplishment.

The following databases were searched for potentially relevant articles, followed by screening the reference lists of identified articles: CINAHL Plus, Embase, MEDLINE, PsycINFO, and Scopus. The study eligibility criteria and selection are outlined in Supplementary Appendix S1. Details pertaining to the search terms, inclusion and exclusion criteria, and search strategy used for each database are also outlined in Supplementary Appendix S1. The review followed the Preferred Reporting Items for Systematic Reviews and Meta-Analyses (PRISMA) guidelines.

### Data extraction

The following data were extracted from each article using a standardised form by one of the reviewers (the first author): geographic location; survey period; sample size with response rate; average age of participants (GPs); number and proportion of male participants; average number of years the participants have worked in general practice; practice size; number of hours worked per week; version of MBI-HSS instrument used to measure burnout; cut-off criteria to denote subcomponents of burnout (emotional exhaustion, depersonalisation, and personal accomplishment) and overall burnout (defined using the criteria used in the study); and mean and proportion estimates of subcomponents of burnout and overall burnout for all the GPs and for male versus female GPs.

### Risk of bias and quality assessment

The risk of bias of the included studies was assessed by one reviewer (the first author) using the Joanna Briggs Institute (JBI) Critical Appraisal Checklist for Studies Reporting Prevalence Data, which scored studies based on nine items that assessed quality. This checklist is described in Supplementary Appendix S2. Full details of the scoring method used and the quality appraisal results for the studies included in this review are provided in Supplementary Appendices S2–S4.

### Pooled analysis

A meta-analysis of high-quality studies, defined using a threshold of seven out of nine items (77.8%) that satisfied the respective quality criteria pertaining to the JBI checklist, was conducted. Stata statistical software (version 16.0) was used to obtain pooled burnout estimates. The meta-analysis commands used are summarised in Supplementary Appendix S5. Pooled mean estimates of the burnout subscales were computed using the metan command for means and standard error, with the standard errors having been calculated in advance using the standard deviations. Prevalence estimates (rates) were computed from these numbers using the metaprop command, reflecting the pooled proportion of GPs who were reported to have experienced burnout. Accounting for potential heterogeneity across studies, a random-effects model was employed to estimate variances of the raw proportions or means.

## RESULTS

### Study characteristics

**Figure 1.**
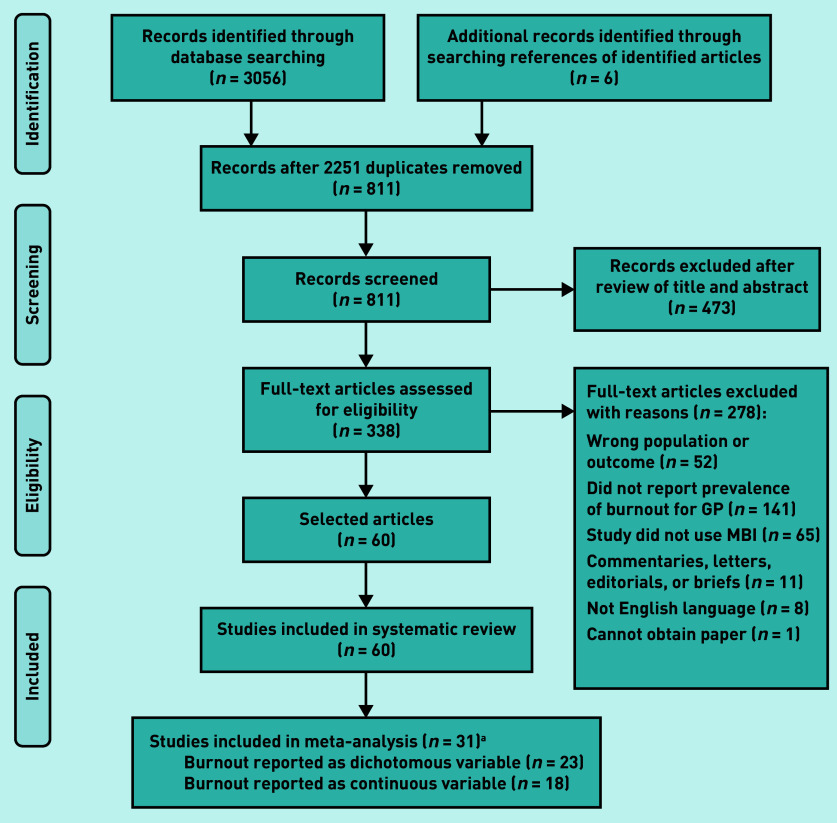
***PRISMA flow diagram on identification and selection of articles.*** *^a^***n*-values are greater than 31 because studies can report burnout as both a dichotomous and continuous variable. MBI = Maslach Burnout Inventory — Human Services Survey.***

The PRISMA flow diagram detailing the selection process for the 60 articles included in the systematic review^25^^–^^84^ is given in Figure 1. Thirty-one of the 60 (51.7%) identified studies met the threshold of ‘high quality’.^31^^,^^33^^,^^35^^,^^38^^–^^40^^,^^42^^,^^43^^,^^45^^,^^47^^–^^49^^,^^51^^–^^56^^,^^58^^–^^63^^,^^65^^,^^70^^,^^72^^–^^75^^,^^83^ Of these studies, 74.2% (*n* = 23/31) reported the number of GPs that had high or moderate burnout along ≥1 of the burnout subcomponents (emotional exhaustion, depersonalisation, and personal accomplishment) and overall burnout; 58.1% (*n* = 18/31) reported mean and standard deviation estimates for ≥1 of the burnout subcomponents (data not shown).

Supplementary Appendix S6 provides a description of selected demographic data extracted from the 60 included studies in this review; burnout cut-offs, mean, and proportion estimates are provided in Supplementary Appendices S7 and S8. Estimates are provided separately for male and female GPs if they are reported in the respective study.

Study time periods ranged from 1987 to 2020, comprising data from 22 177 GPs across 29 countries spanning five continents. The majority of these studies (70.0%, *n* = 42/60) were conducted in Europe, 18.3% (*n* = 11/60) were conducted in Asia, with the remaining studies conducted in the following three continents: Africa 1.7% (*n* = 1/60), North America 3.3% (*n* = 2/60), and Oceania 6.7% (*n* = 4/60). Where a study was conducted over different time periods, data for the earliest period were extracted (Supplementary Appendix S6). Most of the studies (70.0%, *n* = 42/60) used the 22-item version of the MBI-HSS (Supplementary Appendix S6).

The studies predominantly used the following standard cut-offs^19^ to denote high burnout for the three burnout subscales: emotional exhaustion ≥27 (38.3%, *n* = 23/60), depersonalisation ≥10 (30.0%, *n* = 18/60), and personal accomplishment ≤33 (28.3%, *n* = 17/60) (Supplementary Appendix S7). As for high overall burnout, the studies (28.3%, *n* = 17/60) generally used the following criteria: high emotional exhaustion and depersonalisation, and low personal accomplishment.

The reported findings collectively show that there is wide variation in the demographic data, as well as burnout cut-offs and estimates, extracted from the studies included in the review. Selected demographic characteristics reported in the 31 high-quality studies are provided in Supplementary Appendix S9. The heterogeneity in demographic and burnout data observed for the 60 included studies remained for the higher-quality 31 studies included in the meta-analysis. However, the ranges of the burnout estimates reported in these studies are considerably narrower than those reported for all 60 studies.

### Pooled results

**Figure 2.**
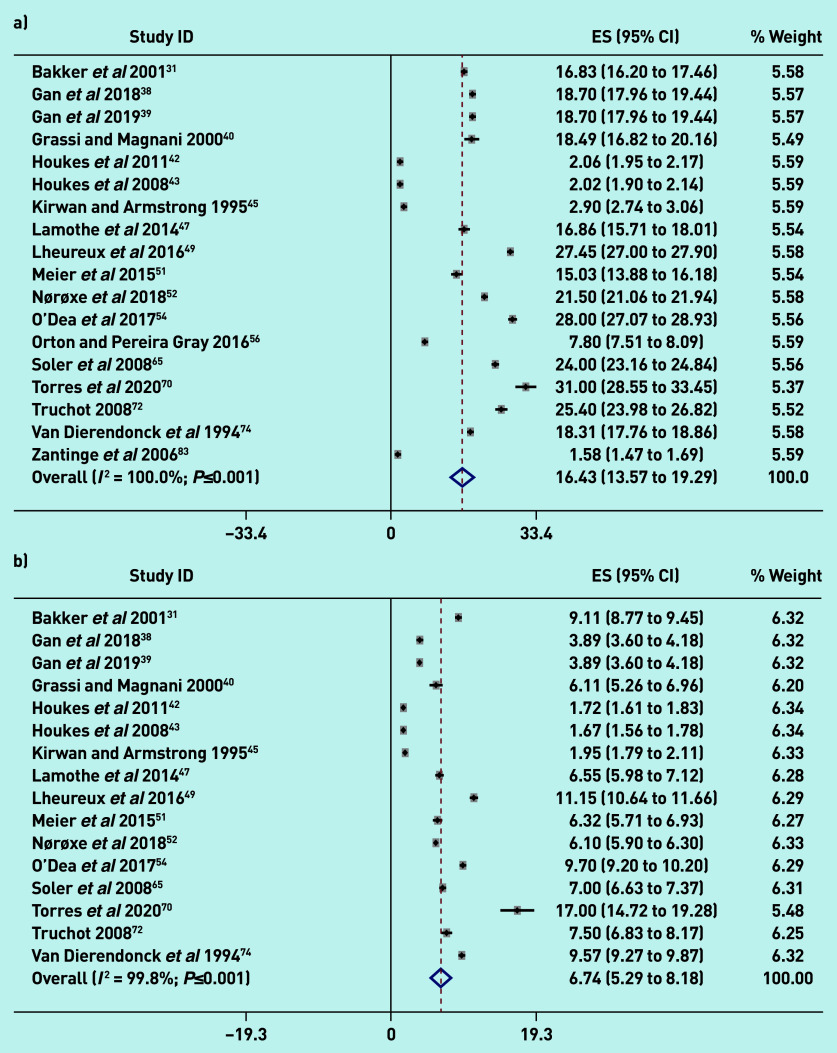

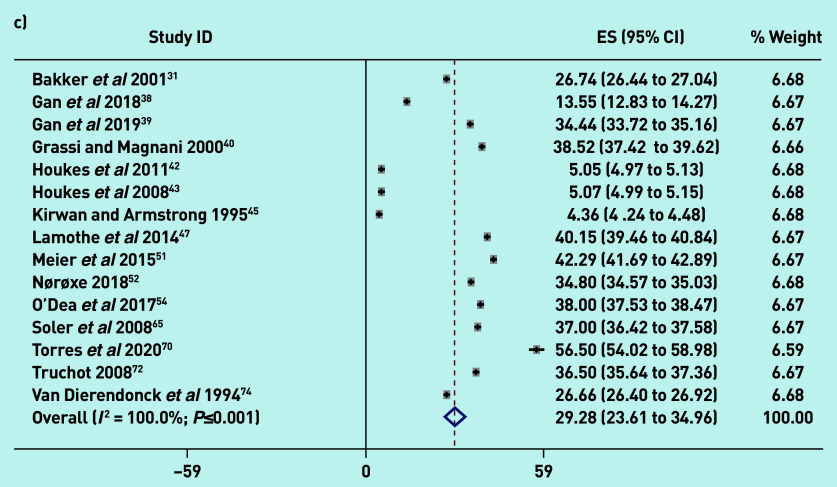
***Meta-analysis of GP burnout using continuous data: a) emotional exhaustion; b) depersonalisation; and c) personal accomplishment. Weights are from random-effects analysis.*** *^a^* *^a^* ***Additionally, while the 31 studies comprised the total set of studies on which the meta-analysis was conducted across all dimensions of burnout, some types of estimates were not reported in some studies. Some studies reported only proportions and/or percentages whereas others reported only mean estimates, and yet others reported both proportions and mean estimates. The total number of studies is 31, which would be reflected by all the studies captured in Figure 2 and also Supplementary Appendix S11. ES = mean score.***

Figure 2 reports the pooled random-effect mean estimates using continuous data based on the scores obtained for the difference burnout subscales: 16.43 (95% confidence interval [CI] = 13.57 to 19.29; *I*^2^ = 100.0%; *P*≤0.001) for emotional exhaustion; 6.74 (95% CI = 5.29 to 8.18; *I*^2^ = 99.8%; *P*≤0.001) for depersonalisation; and 29.28 (95% CI = 23.61 to 34.96; *I*^2^ = 100.0%; *P*≤0.001) for personal accomplishment.

These estimates denote moderate levels of burnout for emotional exhaustion and depersonalisation, and a high level of burnout for personal accomplishment, based on standard burnout cut-offs for these subscales, indicating significant levels of burnout among GPs. As evident in the high *I*^2^ (>99%), there is considerable heterogeneity across studies. Supplementary Appendix S10 shows that the mean burnout estimates for the different burnout subscales varied depending on the country’s geographical region (*P*-value for heterogeneity ≤0.001). Meta regressions results showed that the continent in which studies were conducted had no effect on variation in mean burnout estimates across studies. There were insufficient observations within subgroups to conduct meta regressions for country. Overall, there was no evidence that the geographical region influenced variation in mean burnout estimates across studies.

Studies reported the following pooled prevalence estimates for GPs that exceeded the threshold for high or moderate burnout (Supplementary Appendix S11): high emotional exhaustion 32% (95% CI = 26 to 39; *I*^2^ = 97.95%; *P*≤0.001); high depersonalisation 31% (95% CI = 19 to 43; *I*^2^ = 99.49%; *P*≤0.001); low personal accomplishment 27% (95% CI = 22 to 32; *I*^2^ = 96.86%; *P*≤0.001); high overall burnout 6% (95% CI = 4 to 9; *I*^2^ = 95.42%; *P*≤0.001); moderate emotional exhaustion 28% (95% CI = 22 to 35; *I*^2^ = 95.79%; *P*≤0.001); moderate depersonalisation 23% (95% CI = 15 to 31; *I*^2^ = 97.55%; *P*≤0.001); moderate personal accomplishment 33% (95% CI = 22 to 44; *I*^2^ = 98.51%; *P*≤0.001); and moderate overall burnout 32% (95% CI = 19 to 44; *I*^2^ = 99.40%; *P*≤0.001).

As evident in the high *I*^2^ (>95%), there is considerable heterogeneity across studies. The results (in Supplementary Appendix S10) of subgroup analyses conducted with at least 10 studies to investigate this heterogeneity show that the prevalence of burnout dimensions varied depending on the country’s geographical region and cut-off for moderate overall burnout (*P*-value for heterogeneity ≤0.001). Although some covariates were dropped because of collinearity, meta regressions conducted using the metareg command showed that the continent in which the studies were conducted was generally not an important determinant of high or moderate burnout (*P*>0.20); however, high depersonalisation was significantly lower in Europe (regression coefficient −0.565; 95% CI = −0.768 to −0.362; *P*≤0.001) and North America (regression coefficient −0.354; 95% CI = −0.646 to −0.063; *P*≤0.001) compared with Asia, and moderate overall burnout was significantly lower in Europe (regression coefficient −0.424; 95% CI = −0.803 to −0.046; *P* = 0.03) compared with Asia.

Taken together, the findings indicate that, although the continent in which the studies were conducted is not a robust determinant of GP burnout across studies, there is some evidence that GP burnout is lower in Europe and higher in Asia.

The subgroup analysis by country revealed that the country the study was conducted in did not influence high emotional exhaustion; high depersonalisation was significantly higher in China (regression coefficient 0.543; 95% CI = 0.386 to 0.700; *P*≤0.001) than in the other countries included in the meta regression; low personal accomplishment was significantly higher in China (regression coefficient 0.213; 95% CI = 0.088 to 0.339; *P* = 0.01), Denmark (regression coefficient 0.220; 95% CI = 0.117 to 0.324; *P*≤0.001), and England (regression coefficient 0.211; 95% CI = 0.080 to 0.341; *P* = 0.01) than in other countries. Overall, there is some evidence that GPs from China experienced higher depersonalisation than GPs from other countries (Supplementary Appendix S10).

In addition, overall, there was high residual heterogeneity for high burnout (≥95% for continent and ≥70% for country) and moderate burnout (≥84% for continent) There was no residual heterogeneity (0.00%) and high explained between-study variance for the cut-off for moderate overall burnout (adjusted *R* ^2^ 99.93%), indicating that this cut-off may be an important determinant of heterogeneity in moderate overall burnout estimates across studies. The findings also reveal that less restrictive burnout criteria used in the studies are associated with higher GP burnout prevalence. For example, the more restrictive criteria for moderate overall burnout used in the studies of high emotional exhaustion and/or high depersonalisation have a smaller regression coefficient of 0.170 compared with the less restrictive criteria of high emotional exhaustion and/or high depersonalisation and/or low personal accomplishment, which has a regression coefficient of 0.355 (Supplementary Appendix S10).

Tests of publication bias via funnel plots^85^ and Egger tests^86^ were conducted and results provided in Supplementary Appendix S12. The results provide no evidence of publication bias using the dichotomous data. Visual inspection of the funnel plots showed no asymmetry in all distributions for burnout studies. Furthermore, the Egger tests did not show significant results and thus suggested no evidence of publication bias among the studies on burnout proportions. However, Egger tests on studies using the continuous data showed some evidence of possible small-study effects, with significant results (*P*≤0.001) for mean emotional exhaustion, mean depersonalisation, and mean personal accomplishment.

As another sensitivity test, the meta-analysis was conducted including studies of lower quality (rated ≤6 on the JBI) that were more susceptible to risk of bias. The results (Supplementary Appendix S13) showed that the burnout estimates were similar and still displayed significant heterogeneity for all studies (including those of lower quality) as for only higher-quality studies.

## DISCUSSION

### Summary

The 60 studies included in this systematic review reported a wide range of demographic characteristics, burnout cut-offs, and prevalence estimates. Some studies characterised burnout as uni- or bi-dimensional, although the vast majority of studies characterised burnout as multidimensional. Other studies contribute to the ambiguity with how burnout is characterised by partitioning burnout into high, moderate, and low dimensions, or using different labels (for example, ‘severe’, ‘high’, ‘extreme’, ‘full’, or ‘complete’ were used to denote high burnout). These variations across studies were observed despite narrowly focusing on only one burnout instrument, the MBI-HSS, and one specialty, general practice.

In the present study there appears to be some evidence that the country the study was conducted in may influence this heterogeneity. It is conceivable that different national cultural factors (for example, general practice being perceived as a calling versus a profit-making enterprise) may influence how workload is perceived and thus burnout experienced by GPs. Furthermore, the different features of the primary care system across countries may influence the GP’s work environment, which in turn may influence the likelihood of burnout. This review has provided evidence that the cut-offs used to denote burnout play an important role in influencing GP burnout estimates across studies. The more restrictive the burnout criteria used, the lower the burnout estimate reported across studies.

### Strengths and limitations

This study is, to the authors’ knowledge, the first to undertake a systematic review and meta-analysis of studies on the prevalence of GP burnout worldwide. Another strength of this study is that it attempted to conduct a rigorous examination of the burden of GP burnout worldwide based on a clearly defined concept of burnout using the MBI-HSS, and focusing only on general practice.

This study, however, has several limitations. First, the studies included in this review were not conducted concurrently. Hence, the findings may be subject to different interpretations across different time periods. Second, the different demographics, at the GP and other levels, across the studies may have influenced how burnout is perceived, and may in turn influence the generalisability of the findings.

Third, although every attempt was made to select studies that were similar in their methodological approach for the quantitative analysis, several differences in the study design remained and reduced comparability across the studies.

Fourth, given this review’s focus on studies using the MBI-HSS, the insights derived in this review should be interpreted with caution, especially given the criticism some researchers have directed toward the MBI-HSS instrument and who have used other instruments such as the Oldenburg Burnout Inventory and the Copenhagen Burnout Inventory. Related to this, the MBI-HSS is subject to criticism of bias generated by self-ratings by responders on the questionnaire used in the assessed studies. To focus narrowly on burnout, studies on constructs related to burnout, such as psychological or occupational stress, were not included in the review. To the extent that these studies also capture GP experiences similar to burnout, this review could be criticised as ignoring a vast literature that may be relevant. In a similar vein, what constitutes burnout has been debated in the literature, and the literature that conflates burnout and depression was excluded. It is conceivable that there is an important overlap between GP mental health, psychological distress, and burnout. More importantly, burnout may be more a manifestation of the GP’s underlying mental condition than solely as a result of the workplace context. Hence, the generalisability of this review’s findings beyond studies using only the MBI-HSS could be called into question. Related to this, this literature may also include articles on burnout using the MBI-HSS that may not have been identified in the search strategy used in this systematic review. The MBI-HSS, used in this review, was designed to capture burnout associated with interpersonal relations. However, GP burnout also arises as a result of factors external to human relations such as workload and electronic documentation. Thus, the MBI-HSS may not fully capture GP burnout.

Fifth, studies conducted in a language other than English were not included, which may limit this review’s generalisability to other studies not conducted in English.

Finally, this review only considered peer-reviewed publications and did not consider published data from non-peer-reviewed outlets, which also may have introduced another type of selection or publication bias.

### Comparison with existing literature

The wide ranges in burnout estimates reported in this review are consistent with those reported in two recent systematic reviews on the prevalence of physician burnout across a range of specialties.^87^^,^^88^ The evidence provided in these studies and the present study may reflect the heterogeneity across studies in the criteria used to define and measure burnout, and thus highlight the importance of uniformity in how burnout is measured and defined across studies.

### Implications for research and practice

This study has shown that the approaches used in prior studies to characterise and operationalise GP burnout are inconclusive, with the reported wide-ranging prevalence estimates possibly influenced by a range of factors, such as using different measurement scales, differing cut-off points to define burnout, differing approaches to how burnout is characterised, and different cultural attributes across countries. An implication of this finding for research, practice, and policy pertaining to addressing GP burnout is that assessing and addressing the syndrome should be undertaken by considering the context GPs work in.

The work environment is challenging for the GP, as the GP’s decisions and actions are influenced by those of the patients and other agents that operate within the primary care system who may have different expectations and demands.^22^^,^^89^ These differences in values and priorities between the GP and other individuals in the primary care system can result in difficult interactions between the GP and these individuals. Additional research on the reasons for high/moderate burnout was beyond the scope of this study, but could be related to differences in priorities between the individual GP and the practice the GP is employed at. For example, the emphasis on efficiency could be perceived by GPs as being at the expense of patient welfare, leading to a potential mismatch in values between the practice and the GP. This could interact with the work-related burden imposed on the GP, perhaps exacerbating the level of burnout.

Recent studies have shown that the COVID-19 pandemic has also played an important role in influencing physician burnout. For example, one study showed that infection or death from COVID-19 among colleagues or relatives showed significant association with higher emotional exhaustion and lower personal accomplishment.^90^ Two other studies reported that GPs described feeling more stressed during the pandemic than they had been previously because of the higher workload (for example, as a result of new responsibilities such as additional safety protocols, learning new technology, and daily emails for prescriptions).^91^^,^^92^ The extraordinary impact of the COVID-19 emergency on GPs, as frontline medical providers, was in part produced by the uncertainty of the procedures and treatments required and the immediate saturation of hospitals for critical case management. GPs had to respond directly to a large number of requests without clear prevention or screening instruments. At the time of writing, GPs were the foundation of COVID-19 vaccination programmes in several countries and remain heavily involved in administering vaccines, with some even involved in COVID-19 diagnoses, thus increasing their workload even further. Differences across countries in the severity of the disease as well as the resources available and methods used to curb and treat it (including inefficiencies associated with supplying vaccines to GPs), and operating under different primary care systems, are likely to exacerbate the impact of COVID-19 on GP burnout across countries. Probing GP burnout in more detail within the GP’s workplace environment is left for future research.

## Data

The dataset is available upon request.
